# The upper bound of cumulative return of a trading series

**DOI:** 10.1371/journal.pone.0267239

**Published:** 2022-04-28

**Authors:** Can Yang, Junjie Zhai, Helong Li

**Affiliations:** 1 School of Computer Science and Engineering, South China University of Technology, Guangzhou, Guangdong, China; 2 School of Economics and Commerce, South China University of Technology, Guangzhou, Guangdong, China; BeiHang University School of Economics and Management, CHINA

## Abstract

We present an upper bound of cumulative return in financial trading time series to formulate the most possible profit of many trades. The bound can be used to formally analyze the cumulative return varied by the number of trades, the mean return, and transaction cost rate. We also prove and show the validation of the upper bound, and verify the trend of cumulative return is consistent with that of the proposed bound via simulation experiments. Introducing a set of stochastic assessment methodology based on bootstrap into the organization of experimental data, we illustrate the influence on cumulative return from the relationship between the mean of return and transaction cost rate, technical trading rules, and stock indexes. To the best of our knowledge, this is the first to present and prove a bound of cumulative return of a stock trading series in theory. Both theoretical analyses and simulation experiments show the presented bound is a good mathematical tool to evaluate the trading risks and chances using given trading rules in stock trading markets.

## I. Introduction

A cumulative return on an investment is the aggregate amount that the investment has gained or lost over time by many trades. A trade series refers to the chain of buy and sell trading events on a stock during a period of time. The main goal of stock investments is to achieve big cumulative return, but not big return of one trade. The motivation of investors participating stock trading is to obtain value-added profit through many times via buy and sell trades. How much can you earn on earth after you buy and sell a stock for many times? We focus on studying this problem in this paper.

However, the stock investment is often quite risky, for the stock price is always affected by political, macro or micro economic events [1]. To pursue high profits, the professional investors participating in stock trading would like to hire fundamental analysis and/or technical analysis to make decisions in stock markets [2]. Empirical evidence has shown that both fundamental and technical analysis can help to achieve great profits in stock market investment [3]. For lack of enough financial advantage and information superiority, unprofessional individual investors probably become victims when institutional investors manipulate stock price [4]. Unwilling to accept such a fact, some investors try to use technical trading rules to assist their stock trading, since a qualitative analysis of macroeconomics fundamentals is usually subjective and hard to assess [5]. Nevertheless, the effectiveness of technical trading rules still remains controversy [6–12], and the setting of parameters of a certain trading rules can greatly influence on the return. How to choose proper technical trading rules is therefore a significant subject for investors to make profit and avoid risk, which is one of our main objectives in this work on the bound of cumulative return. In this paper, we present an upper bound of the return, which produces a set of new approaches for evaluating the performance of technical trading rules.

The main goal of this paper is to find a kind of mathematical tool to evaluate and analyze the impact of trading behavior on the cumulative return of many trades, but not a new technical trading approach. We therefore present the maximum possible return on n times of stock trading, which can answer to **how much you will earn at most for many trades**. As one of the fundamental concerns in stock markets, the related literature to the cumulative return is extensive [7, 8], especially on cross-sectional return [13], but there is rare published work on the bound of cumulative return in trading time series. Although using machine learning [14–16] and deep learning [17–21] for stock trading prediction is not the focus of this paper, the proposed bound can be used for studying and evaluating them as well as similar scenarios as portfolio optimization [22, 23], where we could model the portfolio of multiple selected stocks as a customized index, and it is very interesting and left for us to study on the theoretical and statistical simulation in future. As for the bound of the cumulative return, prior work is often based on pricing of assets, but seldom focuses on trading behaviors. For example, [24] provided the theoretical upper bounds, and found the empirical *R*^2^*s* are more than theoretical upper bounds on predictive regression. Similarly, an important bound on cumulative return on Mean-Variance model was earlier studied by [25], which is focusing on is portfolio selection. Different from previous work, we propose an upper bound of cumulative return to evaluate and control the trading risk and profit by selecting proper trading rules in investment. The bound can be also used to formally analyze the cumulative return varied by the number of trades, the mean return, and transaction cost rate.

In this work, we first formally present an upper bound of the cumulative return, and then theoretically prove it. Using bootstrap methodology, we also investigate the effectiveness of the bound in evaluating the performance of technical trading rules on both emerging market and developed market. In short, our study makes several contributions as follows:

1. We presented and proved the upper bound of the cumulative return of stock trading, which paves a new way for formally analyzing maximal possible profit of a stock trading time series.
2. Based on the presented bound, we found and proved preliminary principles on the return of stock trading, and provided mathematically evidence for some trends and laws in stock trading fields.
3. By simulation experiments, we found the moving trend of the bound is basically ordinance with that of the trading return, which can therefore be used for evaluating the performance of trading strategies.

The rest of this paper is organized as follows. Section II first presented the theoretical model on the upper bound of the cumulative return in general stock trading behaviors, and then prove the bound and its relevant corollary. Section III introduced experimental dataset, methods, used technical rules, results and some analyses. Finally, Section IV made our conclusions and prospected the forthcoming work.

## II. The upper bound of cumulative return

To present the upper bound of cumulative return, we first formulate the mathematical model on the general stock trading activities. The symbols used in this section are listed in Table 1.

**Table 1:** Symbols and notations.

Symbol	Notation	Expression
i	Index of a trade	
*P*	Price of a time series of stock trading	
*P*(*b*_*i*_)	The Buying Price of the i-th stock trade	
*P*(*s*_*i*_)	The Sale Price of the i-th stock trade	
*r*_*i*_	The return from the i-th stock trade	Eq (1)
n	The number of trading a stock	
R(n)	The Cumulative Return after undergoing n trades	Eq (2), Eq (3), Eq (4)
*Cost*_*i*_	The trading cost of the i-th trade, e.g., tax and commission, etc.	
*k*	The transaction cost rate of each trade	
$\bar{r}$	The simple average return of n trades	
*UB*(*n*)	The upper bound of cumulative return after undergoing n trades	Eq (5)

Assume P is a time series of stock trading, the return of the i-th trade, in no consideration of transaction cost, is defined as,

$$r_{i}=\frac{P(s_{i})-P(b_{i})}{P(b_{i})}$$ (1)

Here, *P*(*) represents the stock price at a given time *, here, *s*_*i*_ denotes the time of i-th buy event, and *b*_*i*_ denotes the time of i-th sell event.

In consideration of transaction costs, the cumulative return of n trades R(n) can be formulated by:

$$R(n)=\prod_{i=1}^{n}(1+\frac{P(s_{i})-P(b_{i})-{Cost}_{i}}{P(b_{i})})$$ (2)

Here, n is the number of trades, and *Cost*_*i*_ represents the transaction costs in the i-th trade, which can be approximately considered as *Cost*_*i*_≈*k*∙*P*(*s*_*i*_), and here *k* denotes transaction cost rate. Why the approximation of *Cost*_*i*_ is only related to the sell price but not taken the buy price is that the fore is much more than the latter in real stock market, referring relevant rules of trading cost to [26].

To simplify the model of cumulative return, we regarded *k* as a constant in this paper, and can obtain:

$$R(n)=\prod_{i=1}^{n}(1-k)\frac{P(s_{i})}{P(b_{i})}$$

Furthermore, to let $\frac{P(s_{i})}{P(b_{i})}\;be\;(1+r_{i})$ by Eq (1), and substitute it into the above Equation, we can obtain:

$$R(n)=\prod_{i=1}^{n}\left(1-k)(1+r_{i}\right)$$ (3)

Based on the above model, we present theorem 1 to formulate the upper bound of R(n) defined as Eq (3).

**Theorem 1 (Upper bound)** ∀*n*>0, ∃*R*(*n*) satisfies the following inequality:

$$R(n)\leq {\left[\left(1-k)(1+\bar{r}\right)\right]}^{n}.$$ (4)

Here $\bar{r}=\frac{1}{n}\sum_{i=1}^{n}r_{i},\;k$ is the transaction cost rate, the upper bound is noted as UB, i.e.,

$$UB(n)={\left[\left(1-k)(1+\bar{r}\right)\right]}^{n}.$$ (5)

To prove the upper bound is correct, we need to prove the **inequality (4).** Before proving UB, we first introduce preliminary knowledge on D-I inequality as follows, which is proposed and proved by Dragomir and Ionescu [27].

### D-I inequality:

*Let f*:*I*⊆*R*→*R be a differentiable convex function on I*, *x*_*i*_∈*I and p*_*i*_≥0(*i* = 1,…,*n*) *with $P_{n}≔\sum_{i=1}^{n}P_{i}>0$. Then we have the inequality as follows,*

$$\frac{1}{P_{n}}\sum_{i=1}^{n}p_{i}f(x_{i})\geq f(\frac{1}{P_{n}}\sum_{i=1}^{n}p_{i}x_{i})$$ (6)

*where f*′(*x*_*i*_) *is the first derivation of f*(*x*) *at x*_*i*_, and $f^{\prime \prime }(x_{i})=\frac{\partial f(x_{i})}{\partial x}$.

Then, using the D-I inequality, we prove the upper bound of cumulative return R(n), i.e., Theorem 1:

### Proof of Theorem 1:

Let *G*(*n*) = −*ln R*(*n*), according to in Eq (*3)*, *then*

$$G(n)=-\ln\prod_{i=1}^{n}\left(1-k)(1+r_{i}\right)=n∙[-\ln\left(1-k\right)]+\sum_{i=1}^{n}\left[-\ln\left(1+r_{i}\right)\right]$$ (7)

In the D-I inequality, we let *p*_*i*_ = 1, so $P_{n}≔\sum_{i=1}^{n}P_{i}=n$, and *f*(*x*) = −*ln x*. It is easily to certify that *f*(*x*) is a differentiable convex function on (0,+∞) because the second derivative of *f*(*x*) is greater than 0 when *x*∈(0,+∞) (see Eq (*8))*:

$$\frac{\partial^{2}f}{\partial x^{2}}=\frac{\partial (\partial (-\ln\;x)/\partial x)}{\partial x}=\frac{\partial (-1/x)}{\partial x}=\frac{1}{x^{2}}>0$$ (8)

Let *x*_*i*_ = 1+*r*_*i*_, *here*, −1≤*r*_*i*_, because the minimum of *P*(*s*_*i*_) *is zero*, *the in Eq (6) can be transformed into*:

$$-n∙\ln\left[\frac{1}{n}\sum_{i=1}^{n}\left(1+r_{i}\right)\right]\leq \sum_{i=1}^{n}\left[-\ln\left(1+r_{i}\right)\right]$$ (9)

*With the assistance of Eq*. *(9) and the Eq*. *(7*), *G*(*n*) *can be further derived:*

$$G(n)\geq n∙[-\ln\left(1-k\right)]-n∙\ln(1+\bar{r})=-\ln{\left[\left(1-k)(1+\bar{r}\right)\right]}^{n}$$ (10)

*here, $\bar{r}=\frac{1}{n}\sum_{i=1}^{n}r_{i}$, and considering that G*(*n*) = −*ln R*(*n*), *we can further obtain*:

$$R(n)\leq {\left[\left(1-k)(1+\bar{r}\right)\right]}^{n}$$ (11)

*Then, the upper bound of R*(*n*) and **Theorem 1** *have been proved*.

**End of proof**.

Here we have proved the upper bound of Theorem 1 using D-I inequality, and there are still other methods to be able to perform the proof of UB, such as AM-GM inequality, Cauchy inequality.

According to UB and **Theorem 1**, we can derive some corollaries about cumulative returns as follows:

**Corollary 1 Zero Limitation of cumulative return**,

$$R(n)\leq 1\;and\;\underset{n\to +\infty }{\lim}\;R(n)=0,\;if\;\bar{r}\leq k$$

**Proof:** *Because* $\bar{r}\leq k$, *therefore*

$$R(n)\leq {\left[\left(1-k)(1+\bar{r}\right)\right]}^{n}\leq {\left(1-k^{2}\right)}^{n}$$ (12)

*because*, *k*∈(0,1), (1−*k*^2^)∈(0,1), *so R*(*n*)<1. *Further*,

$$0\leq \underset{n\to +\infty }{\lim}\;R(n)\leq \underset{n\to +\infty }{lim}\;{\left(1-k^{2}\right)}^{n}=0$$ (13)

### End of proof

**Fig 1:**
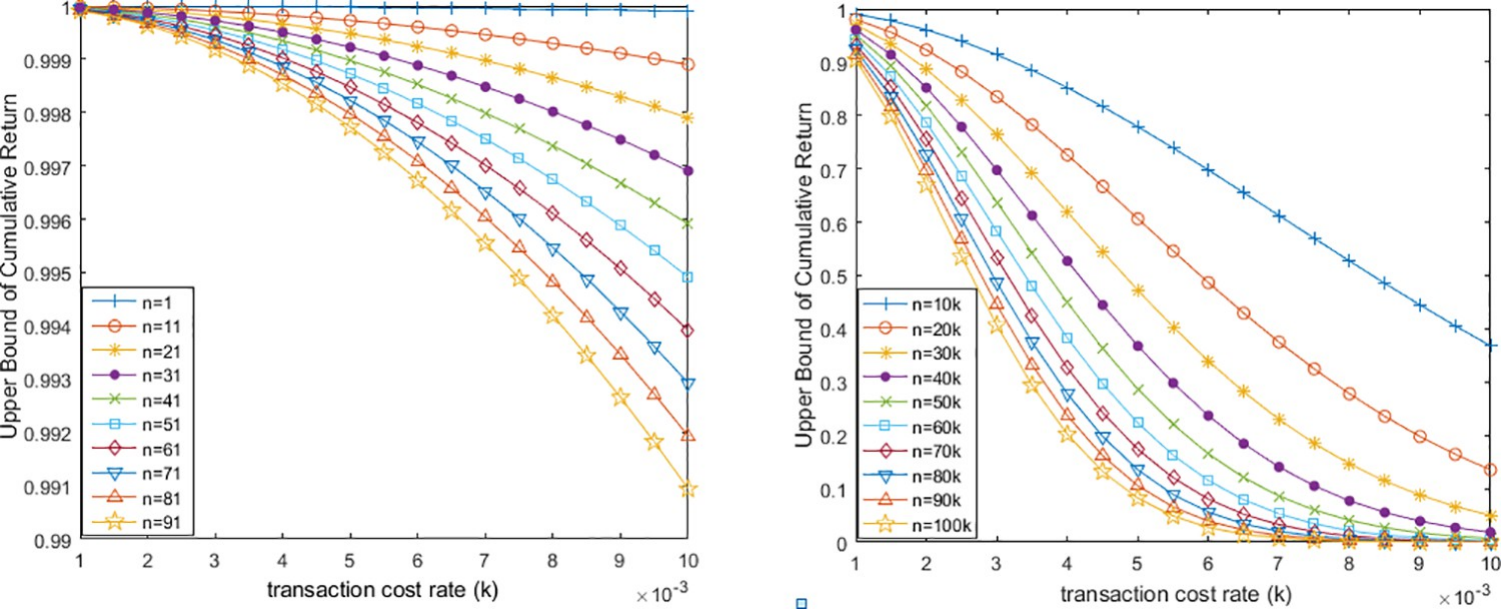
When $\bar{r}\leq k$, the upper bound will decrease with the increase of **k** and **n**. Here the left sub-graph shows the situation when n is small (range from 0 to 100), while the right sub-graph shows the situation that when n is lager enough (range from 10,000 to 100,000). As shown in Fig 1, the upper bound exhibits a clear decreasing trend with the n and k increase.

Obviously, **corollary 1** reveals the that when the number of trades (n) increases to infinity, the cumulative returns will converge to 0 if the mean of returns is less than transaction cost rate, i.e., $\bar{r}\leq k$, which means investors will lose all the money after these trades. In addition, we indicate the trend of upper bound with the increase of k and n in Fig 1. Easy to find when $\bar{r}\leq k$, the declining situation of the upper bound with n is small, while when n is large enough, the upper bound would become close to zero. This is in line with Corollary 1.

On the country of corollary 1, the UB will increase with n if the mean of returns is more than transaction cost rate, i.e., $\bar{r}>k.∵UB={\left[\left(1-k)(1+\bar{r}\right)\right]}^{n}\;and\;\bar{r}>k,∴{UB>(1-k^{2})}^{n}\;and\;UB>{\left(1-{\bar{r}}^{2}\right)}^{n},∵\bar{r}>k⟹{\left(1-{\bar{r}}^{2}\right)}^{n}<{\left(1-k^{2}\right)}^{n},∴{UB>(1-k^{2})}^{n}>{\left(1-{\bar{r}}^{2}\right)}^{n}$. From the analysis on UB, we know that the condition of $\bar{r}>k$ would make the cumulative return *R*(*n*) speed up the increase with n.

## III. Simulation experiments

To illustrate the impact of the upper bound on cumulative returns, we design a set of experiments using historical data of stock indices. In this section, we first introduce the used data set, the used technical trading rules, sub-dataset selected by bootstrap methods, then show the validation of the upper bound, and evaluate the performance of used technical indicators for the stock indices.

### A. Data materials

**Fig 2:**
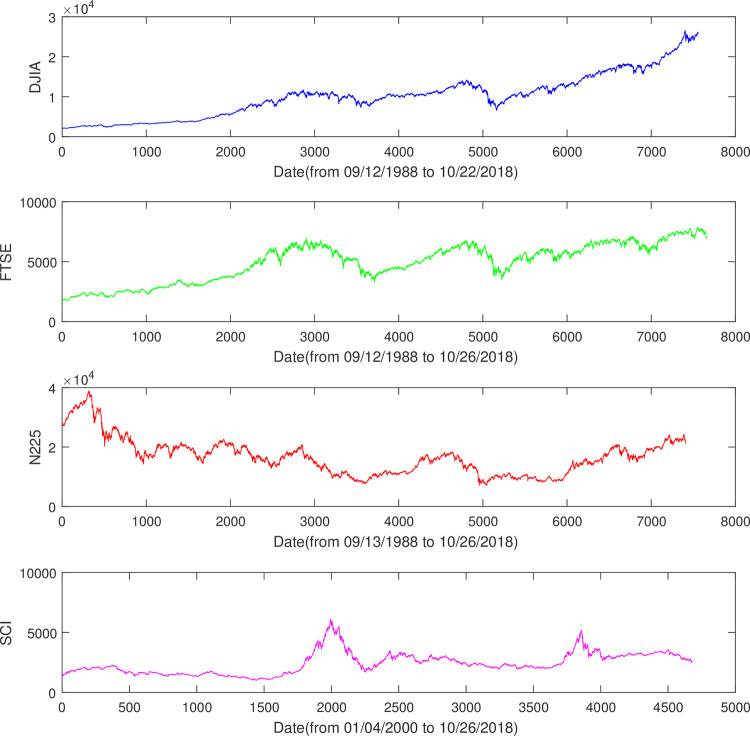
Temporal price movement of four stock indices. From top to bottom, DJIA, FTSE, N225 and SCI indices.

In this paper, the profitability of used technical trading rules is assessed for four important financial market indices, i.e., Dow Jones Industrial Average (DJIA), FTSE 100 Index (FTSE), Nikkei 225 (N225) and SSE Composite Index (SCI). We utilize the approach introduced in [28] and Python package of pandas_datareader to download the daily indices from a popular publicly available source of data, Yahoo Finance [29], where the “Historical Data” of an index can been accessed. Further, we show the price K chart of these stock indices in Fig 2.

- DJIA, from September, 12th 1988 to September, 09th 2018, 7562 days;
- FTSE, from September, 12th 1988 to November, 26th 2018, 7663 days;
- N225, from September, 13th 1988 to November, 26th 2018, 7408 days;
- SCI, from January, 04th 2000 to November, 26th 2018, 5464 days.

### B. Technical trading rules

It is well-known that technical trading rules are widely used in stock trading markets. In this part, we introduce several the used technical trading rules for our simulation trading experiments, involving moving average convergence divergence (MACD), commodity channel index (CCI), rate of change (ROC), and random trading strategy (RND). As for a simple trading strategy [5], the random trading strategy makes trade decision (buy or sell) at the time (t) in random (here, t follows a uniform distribution with a mean of 15). Except for the random trading strategy, the technical trading rules and their formula are presented in Table 2. Notably, the parameters of these trading rules are configured according to [30].

**Table 2:** Details of technical trading rules.

Indicator	Formula	Parameter	Technical rules	
			Buy	Sell
**MACD**	MACD(m,n)=EMA(n)−EMA(m), $EMA(n)=[\frac{\left(C_{t}-{EMA}_{t-1}\right)}{n+1}]+{EMA}_{t-1}$, Here, EMA refers to Exponential Moving Average, n denotes the number of short duration, and m denotes the number of long duration to compute the average of close prices. *C*_*t*_ represents the close price of current time.	n = 12 days, m = 26 days	↑ 0	↓ 0
**CCI**	$CCI(n)=\frac{M-M(n)}{d(n)\times 0.015},\;M(n)=\frac{1}{n}\sum_{i=0}^{n-1}C_{i},\;M=(H+L+C)/3$, here, C, L, H represent close price, low price, high price, respectively; $d(n)=\frac{1}{n}\sum_{i=0}^{n-1}\left|M_{t-i}-{\bar{M}}_{t}(n)\right|$, here, n also denotes the number of short duration	n = 9 days	↑ 100	↓ 100
**ROC**	$ROC(n)=(\frac{C_{t}}{C_{t-n}}-1)\times 100$, C represents the close price, and subscript t denotes the given moment.	n = 13 days	↑ 0	↓ 0

the arrows, ↑ and ↓ respectively mean upwards(golden)/downwards(dead) cross.

### C. Bootstrap method

To reduce the influence of "luck" and make the results more convincing, we adopt bootstrap methodology [31] for the selection of each time experimental data. The main steps of the used bootstrap method in this paper are described as follows.

1. Resampling for data period: for each one of the experiments, randomly choose entering and exiting points for each simulation trading on the used stock index, forming a test period [*enterpoint*_*i*_, *exitpoint*_*i*_];
2. Generating return series and computing the cumulative return: in each test period, use a certain trading rule to make trades and obtain the corresponding return series, then compute cumulative return *R*_*i*_ by Eq(3);
3. Repeating step 1 and step 2: repeat the above two steps for M times, then get the estimation of $\bar{R},\;\bar{R}=\frac{1}{M}\sum_{i=0}^{M}R_{i}$. In this paper, we let M be 1000.

In this part, using the bootstrap method, we first investigate the validity of the proposed upper bound in different stock indices and then provide a method to assess the performance of trading strategies. Besides, we conduct numerous experiments on distinct stock indices to testify the validity of the proposed method.

### D. Validation of the upper bound

**Fig 3:**
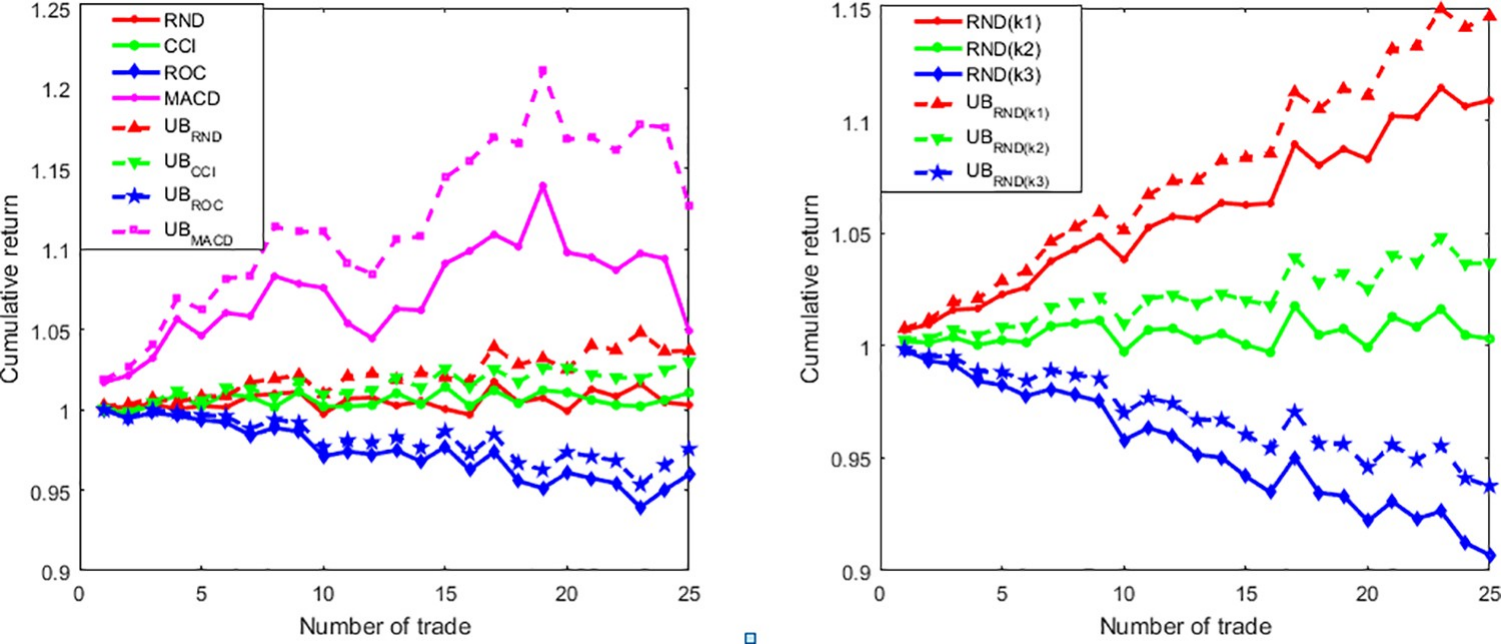
Validation of the upper bound of the cumulative return for DJIA. Here left sub-graph shows the cumulative return and its upper bound with different trading strategies (k = 0.005). While right sub-graph shows the cumulative return and its upper bound with transaction cost rate. k1, k2 and k3 take 0.003, 0.005 and 0.007, respectively. Notably, UB represents the upper bound. As shown in Fig 3, the upper bound can always be held, regardless of trading strategies and transaction cost rate.

To show the validity of proposed upper bound in real stock indices, we choose DJIA as an example to demonstrate the influence of the number of trades on the cumulative return and its upper bound. Fig 3 visualizes the results using the data of **DJIA**, from which we found that in no consideration of transaction cost rate and technical trading rules, the corresponding upper bound can be held, that is to say, the validity of upper bound can be verified to work well in use of the hired index data and technical indicators. The significance of this part is mainly to make a visualization by simulation trades for the proved theorem 1.

### E. Performance evaluation of typical technical indicators

In Section II, we have proved the validity of the upper bound. Here we put forward a method to evaluate the performance of trading strategies. First, we should compute $\bar{r}$ of the trading strategy in the selected stock index. To some extent, $\bar{r}$ can represent the potential profitability of the technical trading rule. Also, we evaluate the performance of trading strategies according to the relationship between $\bar{r}$ and k. If $k>\bar{r}$, the trading strategy will perform poorly for this stock and should be abandoned.

**Fig 4:**
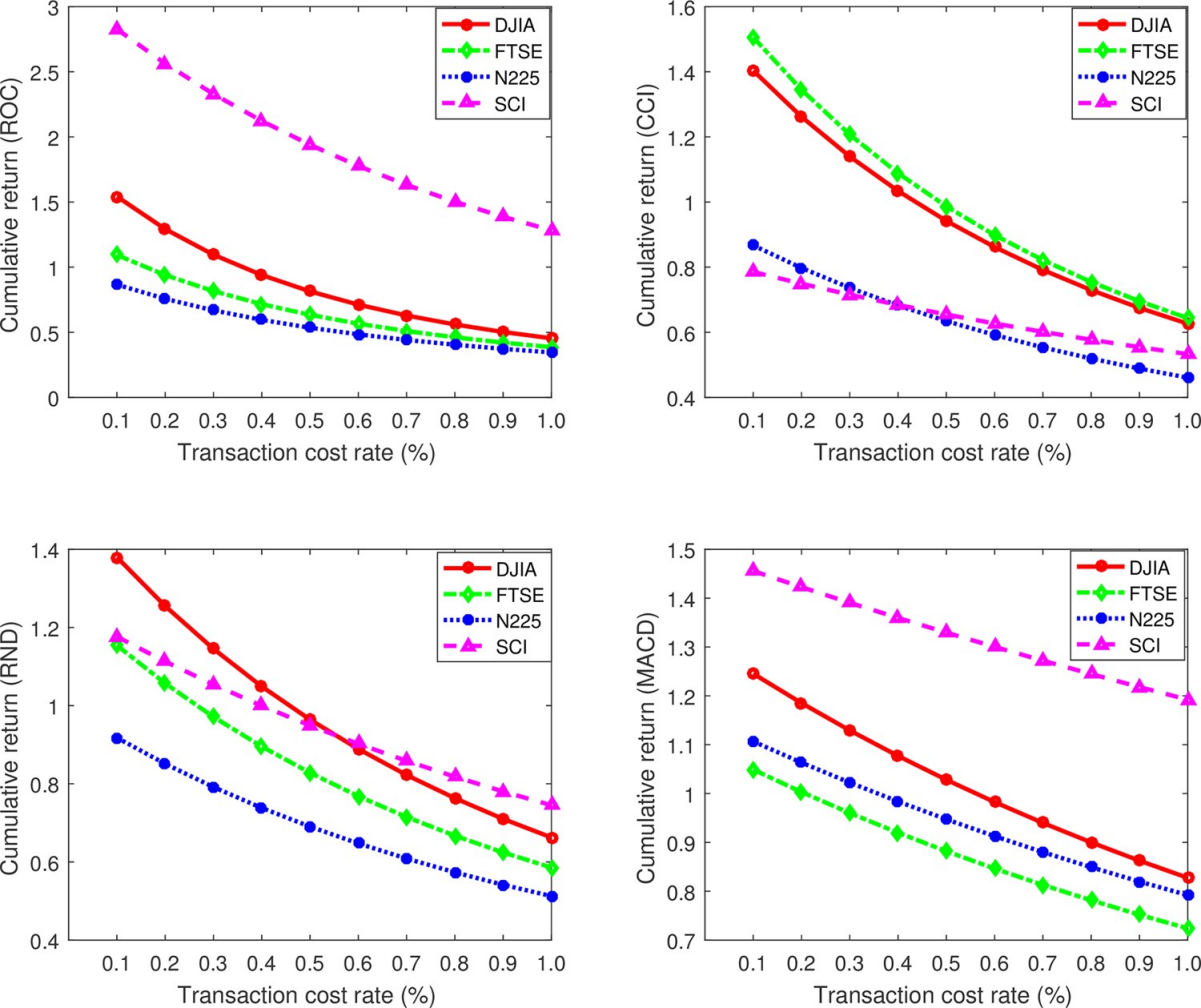
The influence of k on the cumulative return for the stock indices. From top to bottom, from left to right, we show the result of ROC, RND, CCI and MACD strategies, respectively. Further analysis can be seen in text.

Then, we will assess the effectiveness of this method by experiments. We divide each stock index into two equal parts, training set and testing set. By using the bootstrap methodology, we calculate the $\bar{r}$ in training set and regard it as the $\bar{r}$ of this stock index. Table 3 presents the $\bar{r}$ of several technical trading rules in different stock indices. Furthermore, we just try to add transaction costs into the trading model. Transaction cost is a major factor that affects the cumulative return and always consists of two major components: explicit costs and implicit costs, the detailed information can be referred to [32]. In simplicity, we only take explicit costs into consideration and approximately measure transaction costs by transaction cost rate k in this paper. Here we show the influence of k on R(n) in different stock indices, and set the value of k range from 0.001 to 0.01 in Fig 4. Undoubtedly, the cumulative return shows a clear downward tendency when the k increase gradually, this matches our common sense. Note that, if $k>\bar{r}$, the cumulative return will be less than 1. For example, the $\bar{r}$ of ROC in N225 is 0.0005 (see Table 3), which is apparently less than k. The cumulative return will be thus less than 1. Fig 4 sustains our analysis.

**Table 3:** The $\bar{r}$ of the technical trading rules for the stock indices.

Indicator	Stock indices			
	DJIA	FTSE	N225	SCI
**CCI**	0.0046	0.0052	0.0002	-0.0021
**MACD**	0.0065	0.0023	0.0059	0.0251
**RND**	0.0048	0.0028	0.0004	0.0048
**ROC**	0.0038	0.0016	0.0005	0.0149

**Fig 5:**
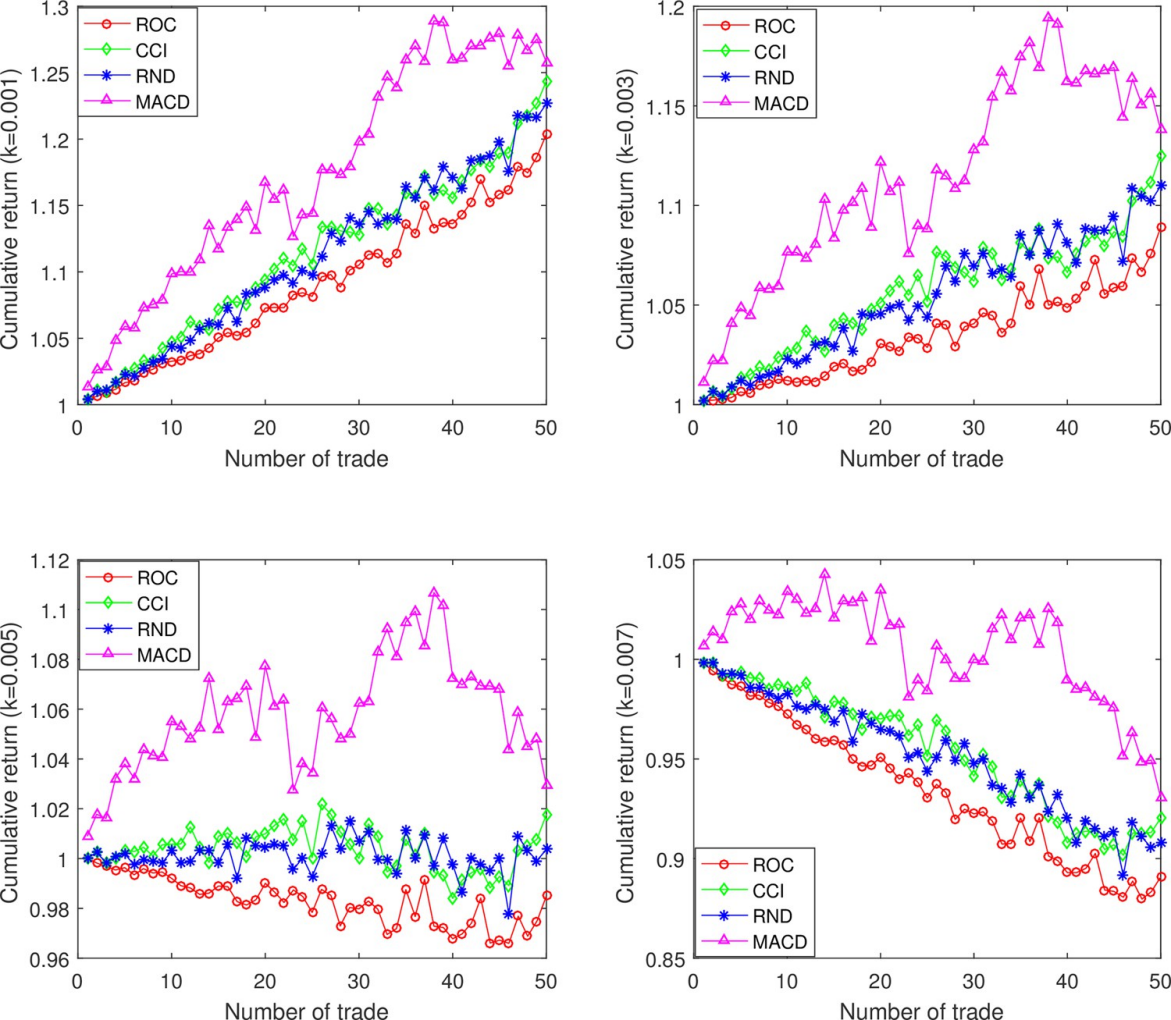
The influence of the number of trades (n) on the cumulative return in DJIA. From top to bottom, from left to right, k takes 0.001, 0.005, 0.003 and 0.007 respectively. See text for additional analysis.

In addition, we also illustrate the influence of the number of trades n on R(n). For simplicity, here we take DJIA as an example and exhibit the results in Fig 5. From Table 3, we know the $\bar{r}$ of the ROC, CCI, RND and MACD for DJIA is 0.0038, 0.0046, 0.0048, 0.0065, respectively. When k takes 0.007, exist $k>\bar{r}$, therefore according to Corollary 1, R(n) ought to show an approximately downward tendency when n increases gradually. Apparently, Fig 5 (k = 0.007) supports our Corollary 1. Anther sub-graph in Fig 5 delivers that if $k<\bar{r}$, it has a large probability that R(n) will increase with the n increases. Therefore, the relationship between k and $\bar{r}$ is a crucial factor in evaluating profitability of a trading strategy in investment. If $k>\bar{r}$, the technical trading rule should not be adopted in the stock index. Note that, the value of k tends to be correlated with the stock market. For example, in the Chinese stock market, the transaction costs mainly consist of stamp duty (0.1% of the turnover), commission (no more than 0.3% of the turnover) and transfer fees (only charge in the Shanghai market, which is 0.006% of the denomination) [12].

In this section, we introduced a set of stochastic assessment methodology based on bootstrap into the organization of experimental data, and illustrate the influence on cumulative return from the relationship between the mean of return and transaction cost rate, technical trading rules, and stock indexes. Besides, the above-mentioned method can be also used for some new methods [7, 8] to evaluate their performance. In prior work, we have studied some deep learning approaches to predict time series, which we are going to plan to use for return prediction if being customized in the future for stock trades. It will be an interesting research direction on how to fusion the bound and deep learning approaches into future stock trading behaviors.

## IV. Conclusions and prospects

In this paper, we first presented an upper bound of the cumulative return for stock trading and proved its correctness in theory. Then we derive and prove a corollary from the upper bound. By the proposed boundary analysis, we interpreted why when the mean of the return rate series $\bar{r}$ less than the transaction cost rate k, the more number of trades, the more loss. Based on the idea of Bootstrap, we furthermore proposed the method to evaluate the performance of trading strategies by using the proposed upper bound. To visualize the validity of the presented bound, using the bootstrap methodology, we conducted numerous simulation experiments to evaluate the performance of technical trading rules.

In short, we conclude that the relationship between k and $\bar{r}$ is a crucial factor in evaluating profitability of a trading strategy in stock markets. Moreover, the proposed bound can be used to control stock trading risk. What’s more, the bound can become one tool of risk control in the high-frequency trading field, for an instance, and be used to the work of [33]. On the other hand, our finding can also be used to help an investor to avoid the unnecessary trades using technical strategies by designing a specific stop-loss mechanism. Both theoretical analyses and simulation experiments showed the presented bound is a good mathematical tool to evaluate the trading risks and chances using given trading rules in stock trading markets.

In the future work, we will pay more attention to combine our finding with other trading strategies assisted by the presented bound, including but being not limited to machine learning, to provide a better guide for investors to control the risk of stock investments.
